# Diffusion Weighted MR Imaging of Primary and Recurrent Middle Ear Cholesteatoma: An Assessment by Readers with Different Expertise

**DOI:** 10.1155/2015/597896

**Published:** 2015-02-02

**Authors:** A. Elefante, M. Cavaliere, C. Russo, G. Caliendo, M. Marseglia, D. Cicala, D. Piccolo, A. Di Lullo, L. Brunetti, A. Palma, M. Iengo, A. Brunetti

**Affiliations:** ^1^Dipartimento di Scienze Biomediche Avanzate-Neuroradiologia, Università Federico II, 80131 Napoli, Italy; ^2^Dipartimento di Neuroscienze e Scienze Riproduttive ed Odontostomatologiche, Università Federico II, 80131 Napoli, Italy

## Abstract

*Introduction and Purpose*. Diffusion weighted imaging (DWI) has been proven to be valuable in the diagnosis of middle ear cholesteatoma. The aims of our study were to evaluate the advantage of multi-shot turbo spin echo (MSh TSE) DWI compared to single-shot echo-planar (SSh EPI) DWI for the diagnosis of cholesteatoma.* Material and Methods*. Thirty-two patients with clinical suspicion of unilateral cholesteatoma underwent preoperative MRI (1.5T) with SSh EPI and MSh TSE. Images were separately analyzed by 4 readers with different expertise to confirm the presence of cholesteatoma. Sensitivity, specificity, diagnostic accuracy, and positive (PPV) and negative predictive values (NPV) were assessed for each observer and interrater agreement was assessed using kappa statistics. Diagnosis was obtained at surgery.* Results*. Overall MSh TSE showed higher diagnostic accuracy and lower negative predictive value (NPV) compared to conventional SSh EPI. Interreader agreement between the observers revealed the superiority of MSh TSE compared to SSh EPI. Interrater agreement among all the four observers was higher by using MSh TSE compared to SSh EPI. * Conclusion*. Our findings suggest that MSh TSE DWI has higher sensitivity for detection of cholesteatoma and lower probability of misdiagnosis. MSh TSE DWI is useful in guiding less experienced observers to the diagnosis.

## 1. Introduction

Middle ear cholesteatoma is a common inflammatory disease requiring prompt surgical treatment to prevent local and intracranial complications due to the activation of osteoclastic function [1]. The diagnosis is generally based on clinical presentation, otoscopic examination, and audiometry. CT scan is used routinely to assess disease extension before surgery. Nevertheless, when the global picture is nonspecific and differential diagnosis is needed, MRI can be used [1]. In particular, diffusion weighted imaging (DWI) can be helpful, since the high content of keratin of cholesteatoma is associated with restricted diffusion [2, 3]. Currently, single-shot echo-planar DWI (SSh EPI) is the most used DWI technique because of its short imaging time (about 2 minutes). On the other hand, multi-shot non-echo-planar DWI sequences (MSh TSE), while requiring a longer imaging time (about 8 minutes), are associated with decreased susceptibility artifacts at the skull base [3–5].

The goal of our study was to compare the diagnostic value of multi-shot non-echo-planar DWI sequences with conventional single-shot echo-planar DWI in patients with clinical suspicion of primary or recurrent middle ear cholesteatoma and to test the interrupter agreement of readers with different expertise levels.

## 2. Materials and Methods

### 2.1. Case Selection

From 2011 to 2013 we conducted a prospective study on a group of 32 patients (18 females and 14 males; age range 11–69 y; mean age 38,9 y) out of 55 in total with clinical and otoscopic suspicion of unilateral middle ear cholesteatoma; 16 patients were suspected of having primary cholesteatoma and 16 of having recurrent disease (residual or relapse). Patients with suspicion of primary or recurrent disease were recruited only when clinical diagnosis was uncertain or differential diagnosis was difficult. All patients with formal contraindications in performing MRI and patients who refuse undergoing MRI or surgery were preliminarily excluded from the study. Between 3 and 7 days before surgery, all patients underwent preoperative MRI to evaluate the extent of the process. The protocol was approved by the local ethical committee and written informed consent was obtained from all patients. An experienced otorhinolaryngologist performed final surgical diagnosis (Table 1).

### 2.2. Imaging Techniques

MRI was carried out at 1.5 T (Philips Intera; Philips Medical Systems; Best, The Netherlands) using an 8-channels head coil. All patients underwent the same protocol, consisting of axial and coronal SE T1w images, axial T2w images, coronal MSh TSE DWI (thickness 3 mm; TE 80.672 ms; TR 3000 ms; FOV 230 × 230 mm; number of average 2; *b* value: 0–1000), coronal SSh SE-EPI DWI (thickness 2 mm; TE 88.364 ms; TR 3774 ms; FOV 230 × 230 mm; number of average 4; *b* value: 0–800), and axial 3D-T2w DRIVE. For each subject we retrospectively calculated diffusion maps (ADC), whose mean value measured 1,19 with SSh SE-EPI and 1,05 with MSh TSE; ADC maps have not been used for the purposes of this study. DWI images with *b* value 1000 s/mm^2^ are positive when the lesion appears hyperintense. For MSh TSE, cardiac gating was applied using peripheral pulse-oximetry to limit artifacts caused by heartbeat and blood flow.

### 2.3. Imaging Evaluation and Statistical Analysis

DWI evaluation was performed by two 10-year experienced neuroradiologists (observers 1 and 2, resp.), who had access neither to patients' clinical data nor to conventional MR findings; MSh-TSE and SSh EPI images were shown to the two examiners separately and in a random order. All readers were asked to express their evaluation by using a dichotomous variable (presence/absence of the middle ear cholesteatoma) defined as a noticeable hyperintensity in the middle ear region. Presence of the lesion was considered as a positive result while absence of the lesion as a negative result, according to the signal characteristics described above. For the two nonexperienced readers the same diagnostic criteria were applied after a brief training sessions where 2 representative cases were shown. ADC map was not shown to the four observers.

Sensitivity, specificity, positive predictive value (PPV), negative predictive value (NPV), and diagnostic accuracy were calculated for each reader and compared with the ones obtained from the other observers. Interrater agreement between the components of each subgroup was assessed by using Cohen's Kappa statistics; interrater agreement among the four observers was assessed by using Fleiss' Kappa statistics.

## 3. Results

**Figure 1:**
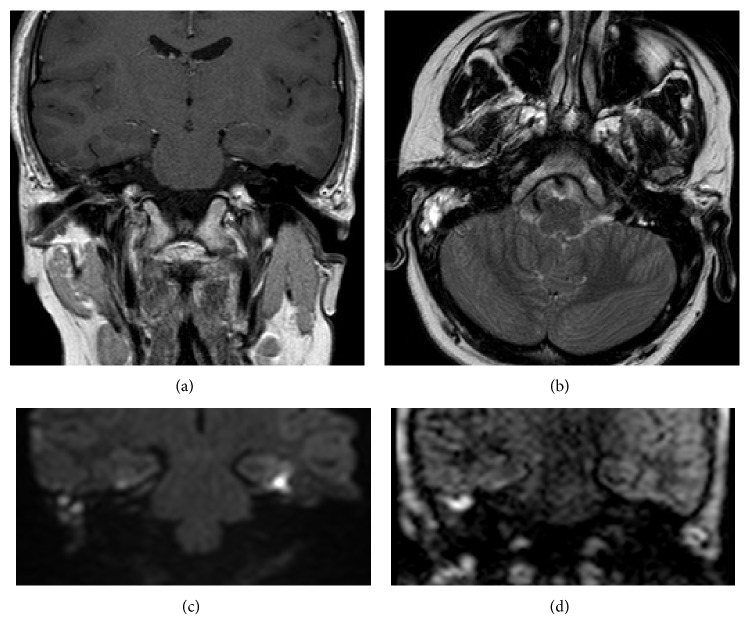
Images of primary cholesteatoma of the right middle ear cavity: (a) coronal SE T1w, (b) axial T2w TSE, (c) coronal SSh SE-EPI DWI (thickness 2 mm; TE 88.364 ms; TR 3774 ms; FOV 230 × 230 mm; number of average 4; *b* value: 0–800), and (d) coronal MSh TSE DWI (thickness 3 mm; TE 80.672 ms; TR 3000 ms; FOV 230 × 230 mm; number of average 2; *b* value: 0–1000).

**Figure 2:**
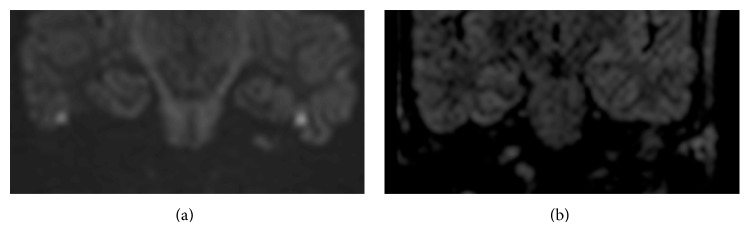
Images of left middle ear cavity in patient with clinical suspicion of primary cholesteatoma: example of SSh SE-EPI DWI false positive (a) compared to MSh TSE DWI true negative (b).

**Figure 3:**
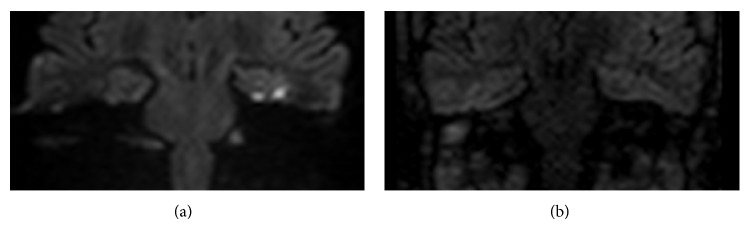
Images of right middle ear cavity in patient with clinical suspicion of recurrent cholesteatoma: example of SSh SE-EPI DWI false negative (a) compared to MSh TSE DWI true positive (b).

**Figure 4:**
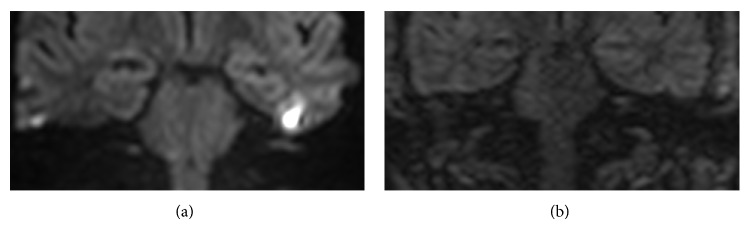
Images of left middle ear cavity in patient with chronic effusive otitis media and suspicion of recurrent cholesteatoma: both coronal SSh SE-EPI DWI (a) and coronal MSh TSE DWI (b) were negatives for cholesteatoma.

The clinical suspicion of middle ear cholesteatoma was surgically confirmed in 24/32 patients (75%). We analyzed the results of the four examiners separately (Figures 1, 2, 3, and 4).

Considering SSh EPI sequences, observer 1 identified 23 true positives, 6 true negatives, 2 false positives, and 1 false negative, while observer 2 identified 19 true positives, 6 true negatives, 2 false positives, and 5 false negatives. Considering MSh TSE image, the first observer identified 24 true positives, 7 true negatives, 1 false positive, and no false negatives and the second observer identified 23 true positives, 6 true negatives, 2 false positives, and 1 false negative.

As regards the two nonexperienced observers, their results were in line with the ones obtained from the two neuroradiologists. Considering SSh EPI sequences, observer 3 identified 18 true positives, 2 true negatives, 6 false positives, and 6 false negatives; observer 4 identified 19 true positives, 2 true negatives, 6 false positives, and 5 false negatives. Considering MSh TSE image, observer 3 identified 22 true positives, 3 true negatives, 5 false positives, and 2 false negatives, while observer 4 identified 19 true positives, 2 true negatives, 6 false positives, and 5 false negatives.

The values measured for sensitivity, specificity, positive predictive value (PPV), negative predictive value (NPV), and diagnostic accuracy are shown in Table 2.

For all the observers MSh TSE showed higher diagnostic accuracy and lower NPV compared to conventional SSh EPI, with fewer incorrect classifications.

Interrater agreement in each subgroup was assessed by using Cohen's Kappa statistics. Interobserver agreement between observers 1 and 2 was considerably higher with MSh-TSE (*K*_Cohen  MSh_ = 87%) compared to SSh SE-EPI (*K*_Cohen  SSh_ = 66%). Stratifying patients by age and sex, no significant difference in interobserver agreement was found; stratifying patients by type of surgical intervention (primary or recurrent cholesteatoma), the interobserver agreement was higher for recurrent cholesteatoma (*K*_SSh  EPI_ = 0.86; *K*_MSh  non-EPI_ = 1) than for primary cholesteatoma (*K*_SSh  EPI_ = 0.59; *K*_MSh  non-EPI_ = 0.86). On the other hand, interobserver agreement between observers 3 and 4 was comparable using MSh-TSE and SSh SE-EPI (*K*_MSh  non-EPI_ = 62% versus *K*_SSh  EPI_ = 65%). Even in this case, stratifying patients by type of surgical intervention, the interobserver agreement was higher for recurrent cholesteatoma (*K*_SSh  EPI_ = 0.86; *K*_MSh  non-EPI_ = 0.66) than for primary cholesteatoma (*K*_SSh  EPI_ = 0.51; *K*_MSh  non-EPI_ = 0.65). No substantial difference in interobserver agreement was brought to light stratifying patients by age or sex.

Interrater agreement among the four above mentioned observers and considering all patients at the same time was assessed by using Fleiss' Kappa statistics. Interobserver agreement was higher with MSh-TSE (*K*_Fleiss  MSh_ = 42%, good agreement) compared to SSh SE-EPI (*K*_Fleiss  SSh_ = 14%, poor agreement) and no significant difference was found stratifying patients by primary/recurrent cholesteatoma, age, or sex.

## 4. Discussion

Recent studies highlighted the ability of diffusion weighted imaging (DWI) to differentiate cholesteatoma from other inflammatory diseases of the middle ear [6]. DWI has shown to have high sensitivity and specificity for detecting cholesteatoma; this is possible because of the peculiar composition of this lesion, whose high keratin content determines a sharp signal hyperintensity in these sequences [3, 4, 7]. The main field of application of this technique is the screening of postsurgical patients, to distinguish residual/recurrent disease requiring second-look surgery from postoperative fibrosis not requiring surgical revision. Hence, DWI can contribute to a significant reduction in the number of second-look surgeries performed, thereby decreasing surgical costs and patient morbidity, with a global advantage in the management of cases of cholesteatoma [7–12]. Since there are many different DWI acquisition sequences, the selection of the most appropriate DWI imaging approach has a potential practical value.

Previous studies demonstrated that, among DWI acquisition techniques, non-echo-planar imaging (EPI) MRI is a more accurate method in detecting middle ear cholesteatoma compared to EPI and it improves the detection of small-sized cholesteatoma and decreases artifacts occurring in the EPI diffusion weighted technique [1, 2, 4, 8, 11, 12]; non-EPI requires also shorter acquisition time in comparison to delayed postcontrast sequences conventionally used in cholesteatoma imaging [2, 4, 7, 8, 13]. In another study it has been demonstrated that MSh EPI improves the diagnostic accuracy of acquired middle ear cholesteatoma compared with SSh EPI [14]. However, even if SSh EPI is the most widely used DWI technique, these procedures can be associated to susceptibility artifacts due to field inhomogeneity in the temporal bone region and this may lead to severe image degradation. In MSh EPI, a longer examination time than SSh EPI is required, but reduced geometric distortions and improved image quality should lead to the use of MSh non-EPI instead of SSh EPI [14].

In our study, with a direct comparison between SSh EPI and MSh TSE, we take a stock of the current situation regarding the most accurate imaging technique in middle ear cholesteatoma diagnosis. Our results are concordant with previous reports supporting the superior value of non-EPI approaches [2, 4, 8]. The diffusion images interpretation by two experienced neuroradiologists showed that, comparing different DWI techniques, MSh TSE increased sensitivity, specificity, and diagnostic accuracy compared to conventional SSh EPI. In MSh TSE DWI, high specificity for keratin-containing lesions is associated with high sensitivity for detection of small lesions. The considerable interrater agreement between experienced neuroradiologists obtained by using Cohen's Kappa statistics, taking into account also the agreement occurring by chance, may suggest that MSh TSE, compared to SSh EPI, can reduce the error rate resulting from the interpretation of DWI images. This is even more critical when considering recurrent middle ear cholesteatoma in patients who have already undergone surgery.

Furthermore, our results suggest that MSh TSE can facilitate the correct diagnosis for neuroradiologists with different levels of expertise in middle ear cavity imaging interpretation. To support our thesis, we decided to administer DWI images to two nonexperienced observers (in our case, two medical students at their last academic year with a good basic technical knowledge); the results obtained were in line with the ones deriving from more experienced observers and, also in this case, MSh TSE was found to have higher sensitivity, specificity, and diagnostic accuracy compared to conventional SSh EPI.

The discrepancy between observers 3 and 4 and observers 1 and 2 regarding sensitivity, specificity, PPV, NPV, and diagnostic accuracy is mainly attributable to the lower level of expertise and to the difficulty in interpreting diffusion images of middle ear cavity. Compared to observers 1 and 2, the interrater agreement between observers 3 and 4 was negatively influenced by the larger number of incorrect interpretations; the majority of these incorrect interpretations are due to the small size (2–5 mm) of the lesion detected in some cases (false negatives) and to the artifacts at the skull base, which could be misinterpreted by observers with limited experience (false positives). However, even in this case the values for agreement were higher considering patients with recurrent cholesteatoma than with primary lesion, for both SSh EPI and MSh non-EPI.

Comparing the results of all the four observers simultaneously by using Fleiss' Kappa statistics, the closeness of the agreement was proved to be superior with MSh TSE compared to with SSh EPI; no significant difference was found stratifying patients by primary or recurrent cholesteatoma and by age or sex. Despite a certain number of discrepancies between the two subgroups of observers due to different levels of expertise, it can be deduced that MSh TSE makes it easier to reach the right diagnosis. From this standpoint it may be concluded that MSh TSE can provide greater support in daily clinical practice compared to SSh EPI, even for less experienced operators.

In conclusion, currently DWI is used to identify suspected recurrent cholesteatoma and to prevent unnecessary second-look surgery [2, 7, 8]. In our experience, MSh TSE can be considered a reliable alternative to conventional SSh EPI for this purpose. The results obtained by nonexperienced readers confirm the potential value of MSh TSE in making the correct diagnosis. In the light of these considerations, we can propose the adoption of an optimized examination protocol for the evaluation of middle ear cholesteatoma. This shorter protocol should be based on a reduced number of MRI sequences, including MSh TSE instead of SSh EPI, with no postcontrast images. MSh TSE brings several advantages, because it is available in most imaging systems, does not require contrast injection, and produces images of easier interpretation for both experienced and nonexperienced observers. The main problems of this technique are susceptibility artifacts at the skull base, motion artifacts due to cardiac movement/blood vessel pulsation, and longer imaging time.

The main limitation of our study is the relatively low number of patients, even if our sample size is in line with the one used in other studies in literature.

## 5. Conclusion

Our results show the superiority of MSh TSE compared to conventional SSh EPI in the study of middle ear cholesteatoma. Therefore, replacement of SSh EPI with MSh TSE in the MRI routine study of primary and recurrent middle ear cholesteatoma because of the increased diagnostic accuracy and the lower NPV, with a substantial reduction of misdiagnosis, could be recommended. We intend to highlight the fact that MSh TSE provides a greater ease in DWI interpretation, which is a crucial factor specially for radiologists not experienced in middle ear cholesteatoma imaging.

## Figures and Tables

**Table 1:** Summary table containing clinical, surgical, and radiological features of patients included in the study, stratified by type of surgical intervention of primary (I) or recurrent (II) cholesteatoma.

No.	Name	Sex	Age	Side	I/II disease	Surgical diagnosis	SSH 1°	MSH 1°	SSH 2°	MSH 2°	SSH 3°	MSH 3°	SSH 4°	MSH 4°
							Operator	Operator	Operator	Operator	Operator	Operator	Operator	Operator
1	B.R.	F	15 aa	sx	I	−	−	−	+	−	+	−	−	+
2	C.A.L.	M	15 aa	dx	I	+	+	+	+	+	+	+	+	+
3	C.M.	M	13 aa	dx	I	+	+	+	+	+	+	+	−	−
4	D.M.M.	M	20 aa	sx	I	−	+	−	−	+	+	−	+	−
5	E.R.	M	46 aa	dx	I	−	+	+	+	+	+	+	+	+
6	F.L.	M	33 aa	sx	I	+	−	+	+	+	+	+	+	+
7	G.S.	M	55 aa	dx	I	+	+	+	+	+	+	+	+	+
8	I.V.	F	45 aa	sx	I	+	+	+	+	+	+	+	+	+
9	L.S.	F	67 aa	dx	I	+	+	+	−	+	+	+	−	−
10	L.C.	F	52 aa	dx	I	−	−	−	−	−	+	+	+	−
11	M.M.	F	38 aa	sx	I	+	+	+	−	+	+	+	+	+
12	P.M.	M	20 aa	dx	I	+	+	+	+	−	−	+	+	+
13	R.P.	M	39 aa	dx	I	+	+	+	+	+	−	+	+	+
14	R.A.	F	27 aa	dx	I	+	+	+	−	+	+	+	−	+
15	S.A.	F	14 aa	sx	I	+	+	+	+	+	+	+	+	+
16	S.A.	M	55 aa	dx	I	−	−	−	−	−	−	+	+	−

1	A.L.	M	62 aa	sx	II	+	+	+	+	+	+	+	+	+
2	C.E.	F	39 aa	sx	II	+	+	+	+	+	−	−	−	−
3	C.C.	F	49 aa	sx	II	−	−	−	−	−	−	+	−	−
4	C.L.	F	22 aa	dx	II	+	+	+	+	+	+	+	+	+
5	D.N.A.	F	69 aa	dx	II	+	+	+	+	+	−	−	+	+
6	D.P.E.	M	11 aa	dx	II	+	+	+	+	+	+	+	+	+
7	I.G.	F	59 aa	sx	II	+	+	+	+	+	+	+	+	+
8	J.G.	F	25 aa	dx	II	+	+	+	−	+	+	+	+	−
9	M.G.	F	48 aa	sx	II	−	−	−	−	−	+	+	+	−
10	M.M.	F	61 aa	dx	II	+	+	+	+	+	+	+	+	+
11	M.M.A.	F	59 aa	dx	II	+	+	+	+	+	+	+	+	+
12	P.C.	F	59 aa	dx	II	+	+	+	−	+	−	+	+	+
13	P.A.	F	30 aa	dx	II	−	−	−	−	−	+	−	+	−
14	P.F.	F	18 aa	dx	II	+	+	+	+	+	+	+	+	+
15	S.A.	M	37 aa	sx	II	+	+	+	+	+	−	+	−	−
16	V.M.	F	42 aa	sx	II	+	+	+	+	+	+	+	+	+

**Table 2:** Summary table of statistical values resulting from the analysis of data produced by each observer, respectively.

	Sensibility	Specificity	Diagnostic accuracy	PPV	NPV
Observer 1					
SSh SE-EPI	0.96	0.75	0.90	0.92	0.86
MSh TSE	1	0.87	0.97	0.96	1
Observer 2					
SSh SE-EPI	0.79	0.75	0.78	0.90	0.55
MSh TSE	0.96	0.75	0.91	0.92	0.86
Observer 3					
SSh SE-EPI	0.75	0.25	0.625	0.75	0.25
MSh TSE	0.92	0.375	0.78	0.81	0.60
Observer 4					
SSh SE-EPI	0.79	0.25	0.66	0.76	0.29
MSh TSE	0.79	0.75	0.78	0.90	0.55
